# Growth and yield performance of *Pleurotus ostreatus* (Jacq. Fr.) Kumm (oyster mushroom) on different substrates

**DOI:** 10.1186/s13568-016-0265-1

**Published:** 2016-10-04

**Authors:** Zenebe Girmay, Weldesemayat Gorems, Getachew Birhanu, Solomon Zewdie

**Affiliations:** 1 School of Forestry, Wondo Genet College of Forestry and Natural Resource, Hawassa University, P.O. Box 128, Shashemene, Ethiopia; 2 Life and Earth Sciences Institute, Pan African University, University of Ibadan, Ibadan, Nigeria; 3 School of Wildlife and Eco-tourism, Wondo Genet College of Forestry and Natural Resource, Hawassa University, P.O. Box 128, Shashemene, Ethiopia; 4 School of Natural Resource and Environmental Studies, Wondo Genet College of Forestry and Natural Resource, Hawassa University, P.O. Box 128, Shashemene, Ethiopia; 5 National REDD+ Secretariat, Ministry of Environment, Forestry and Climate Change (MEFCC), Addis Ababa, Ethiopia

**Keywords:** Agro-wastes, Biological efficiency, Fruiting body, Yield

## Abstract

Mushroom cultivation is reported as an economically viable bio-technology process for conversion of various lignocellulosic wastes. Given the lack of technology know-how on the cultivation of mushroom, this study was conducted in Wondo Genet College of Forestry and Natural Resource, with the aim to assess the suitability of selected substrates (agricultural and/or forest wastes) for oyster mushroom cultivation. Accordingly, four substrates (cotton seed, paper waste, wheat straw, and sawdust) were tested for their efficacy in oyster mushroom production. Pure culture of oyster mushroom was obtained from Mycology laboratory, Department of Plant Biology and Biodiversity Management, Addis Ababa University. The pure culture was inoculated on potato dextrose agar for spawn preparation. Then, the spawn containing sorghum was inoculated with the fungal culture for the formation of fruiting bodies on the agricultural wastes. The oyster mushroom cultivation was undertaken under aseptic conditions, and the growth and development of mushroom were monitored daily. Results of the study revealed that oyster mushroom can grow on cotton seed, paper waste, sawdust and wheat straw, with varying growth performances. The highest biological and economic yield, as well as the highest percentage of biological efficiency of oyster mushroom was obtained from cotton seed, while the least was from sawdust. The study recommends cotton seed, followed by paper waste as suitable substrates for the cultivation of oyster mushroom. It also suggests that there is a need for further investigation on various aspects of oyster mushroom cultivation in Ethiopia to promote the industry.

## Introduction

Mushroom, a macro fungus with a distinctive fruiting body, is a unique biota which assembles its food by secreting degrading enzymes. It decomposes the complex organic materials on which it grows (the substrate) to generate simpler compounds for its nutrition (Chang and Miles 1992). These substrates are usually by-products from industry, households and agriculture, and are usually considered as wastes. These wastes, if carelessly disposed of in the surrounding environment by dumping or burning, will lead to environmental pollution and consequently cause health hazards. However, they are actually resources in the wrong place at a particular time and mushroom cultivation can harness these resources (wastes) for its own beneficial advantage (World Bank 2004). Mushroom cultivation, which is reported to represent the only economically viable bio-technology process for conversion of waste plant residues from forests and agriculture (Wood and Smith 1987), fits very well into this category. Mushroom cultivation technology is environmentally-friendly; the mushroom mycelia can produce a group of complex extracellular enzymes which can degrade and utilize the lignocellulosic wastes and thereby reducing pollution. Recently, it has been revealed that mushroom mycelia can play a significant role in the restoration of damaged environments (*myco*-*restoration*) through *myco*-*filtration* (using mycelia to filter water), *myco*-*forestry* (using mycelia to restore forests), *myco*-*remediation* (using mycelia to eliminate toxic waste), and *myco*-*pesticides* (using mycelia to control insect pests) (Stamets 2005). These methods represent the potential to create clean ecosystem, where no damage will be left after fungal implementation.

Mushrooms can not only convert lignocellulosic waste materials into human food, but also can produce notable nutriceutical products, which have many health benefits. They provide people with an additional vegetable of high quality, and enrich the diet with high quality proteins, minerals and vitamins which can be of direct benefit to the human health and fitness. Edible mushrooms are highly nutritious and can be compared with eggs, milk and meat. The extractable bio-active compounds from medicinal mushrooms would enhance human’s immune systems and improve their quality of life. The content of essential amino acids in mushroom is high and close to the need of the human body. Mushroom is also easily digestible and it has no cholesterol content (Oei 2003). The spent substrate left after harvesting the mushrooms, which is entangled with innumerable mushroom threads (collectively referred to as mycelia), can also be used as animal feed (more palatable), bio-fertilizer for soil fertility enrichment and biogas (Alice and Kustudia 2004).

Furthermore, mushroom cultivation can be a labor-intensive agro-industrial activity, thus can help generate income and employment, particularly for women and youth in developing countries. Mushrooms are relatively fast growing organisms, thus, mushroom cultivation as a short return agricultural business can be of immediate benefit to the community. While land availability is usually a limiting factor in most types of primary production, mushroom cultivation requires relatively little space; they can be stacked using shelf-like culture systems. It is, therefore, hoped that the avocation of mushroom farming will become a very important cottage industry in integrated rural development programs. This will lead to the economic betterment of not only small-holder farmers but also of landless laborers and other weak sections of communities (Alam and Raza 2001; Sher 2006; Shah et al. 2004; Flores 2006). Generally, mushroom cultivation technology is very vital in the tackle against *shortage of food*, *diminishing quality of human health* and *pollution of the environment*, which human beings still face, and will continue to face, due to the continued increase of the world population, natural resource degradation and impacts from climate change (Oseni et al. 2012; Chang 2008).

Despite such versatile benefits, the mushroom industry in Ethiopia lacks the necessary technology know-how and adoption. Although Ethiopia has favorable climate, comparatively abundant land and labor as well as reasonably good water resources, the production and utilization of mushrooms in Ethiopia has been neglected so far. Consequently, the country has not benefited from mushrooms as the rest of the world (Kiflemariam 2008).

Moreover, the lack of appreciation about the food and dietary importance of mushrooms, and the monotonous traditional diets and the conservative eating habit of Ethiopian people are among the main impediments constraining mushroom cultivation in Ethiopia. As a result, promoting technology transfer concerning to mushroom cultivation is urgently required intervention option. With this rational, the present study was initiated to investigate the suitability of various organic wastes, in this case, sawdust, cotton seed, wheat straw and paper waste on production potential (growth performance and yield) of oyster mushroom. The implication of this study is to facilitate technology adoption of oyster mushroom cultivation using agricultural and/or forest wastes, and thereby identify the feasibility of mushroom cultivation in the study area for the betterment of the life of the local community.

## Materials and methods

### Study area and experimental materials

The study was conducted at Wondo Genet College of Forestry and Natural Resources, Hawassa University from March to July, 2014. Substrate quality of four different substrates namely, sawdust, cotton seed, wheat straw, and paper waste were evaluated for growing oyster mushroom. Sawdust and paper waste were collected from Wondo Genet, cotton seed was purchased from Addis Ababa, and wheat straw was collected from Arsi Negele district. Pure culture of oyster mushroom (*Pleurotus ostreatus* (Jacq. Fr.) Kumm) was obtained from Mycology laboratory, Department of Plant Biology and Biodiversity Management, Addis Ababa University. The culture code of *Pleurotus ostreatus* (Jacq. Fr.) Kumm is M2153.

### Pure culture preparation and production conditions

Mushroom culture was grown on Malt Extract Agar (MEA, Oxoid) medium for 7 days. Fifty gram of MEA was mixed with 1L of distilled water. Test tubes were corked, sterilized (at 121 °C, 1.5 p.s.i, for 30 min) and allowed to solidify in a slant position. To obtain pure culture, a piece (4 mm × 4 mm) of fleshy tissue (of the original *Pleurotus ostreatus* culture) was aseptically transferred to individual MEA slants under UV fitted inoculation chamber. The cultures were incubated at 25 °C until sufficient mycelial growth is observed and pure cultures were obtained by sub-culturing in MEA. The slant culture was transferred to petri-plates and incubated at 25 °C for 7 days. Once the mycelium fully invaded the agar medium, the culture was used for spawn preparation.

### Grain spawn preparation

Sorghum (*Sorghum bicolor* L.) grain was used for spawn preparation. For this purpose, about 3.6 kg of sorghum grain was half-cooked, excess water drained off and allowed to cool down to room temperature. It was then spread uniformly over a surface sterilized (70 % ethanol) plastic sheet until optimum moisture (51–54 %) is attained. The grain was then mixed with 10 % wheat bran, and 2 % gypsum (calcium sulfate) as nutrient supplement and the pH was adjusted to 9 (Romero 2007). The mixture was then filled into sterilized one-liter bottles to two-third capacity, plugged and sterilized in an autoclave (at 121 °C, 1.5 p.s.i, for 1 h). Sterilized bottles were then allowed to cool and aseptically inoculated with a piece (5 mm × 5 mm) of mycelia culture (14 days old). The bottles were subsequently incubated at 24 ± 3 °C for 14 days until the mycelia fully invade the grains. After 15 days, the grain spawn was ready to use.

### Substrate processing and spawning

The substrates were allowed to dry in the sun for 10 days and regularly (daily) weighed for five consecutive days to determine the change in the air dry weight of substrates. One kilogram air dried substrate was transferred to individual polypropylene bags (55 cm × 50 cm). The opening of each bag was tied and soaked with distilled and sterilized tap water overnight. After overnight soaking, excess water was drained off, left in full sunlight and occasionally weighed until the moisture content was brought to 65–70 % (Iqbal et al. 2005). The substrates were sterilized in an autoclave and allowed to cool to room temperature for several hours. Following this, substrates in separate bags were transferred to surface sterilized polythene bags (65 cm length and 45 cm width) and each bag (with 1 kg substrate) was inoculated with 80 g of spawn. For a uniform distribution of the inocula, spawn and substrates were mixed thoroughly under aseptic condition. Several (6–8) holes were punched on the sides of the plastic bags to facilitate cross-sectional ventilation. Finally, a total of 8 polythene bags from each substrate type were inoculated with a spawn and the experiment was done in duplicates.

### Cultivation conditions and cropping system

Following the method of Chang and Miles (2004), inoculated bags were put in a dark locker to initiate mycelia growth. After mycelial growth in the bags became abundant and/or pinheads emerged, portions of the bags were cut-off to create perforations to facilitate the development of fruiting bodies. Then, fully colonized substrates were transferred to growth room and placed on racks (made from wood and nylon rope) at a spacing of 15–20 cm. Proper ventilation of the growth room was assured by opening the door occasionally (every 2–3 days). Inoculated bags were watered 2–3 times a day to keep the mycelia moist. Relative humidity (RH) and room temperature were monitored and maintained with thermo-hygro meter and RH was maintained between 80 and 85 % by spraying fine mist of water occasionally (Oei 2003).

### Data collection and analysis

The growth and development of mushroom were monitored daily. The time (number of days) required from inoculation to completion of mycelium running, time elapsed between opening the plastic bags to pinhead formation and time required from opening the plastic bags to first round harvesting were recorded. Growth parameters including stipe length (cm), stipe diameter (cm), pileus diameter (cm), and pileus thickness (cm) were recorded with a slide caliper before each harvest. Yield parameters, such as number of fruiting bodies per bunch, and total fresh weight (g) of mushroom were also recorded at harvest time. Matured fruiting bodies (white in color, with up curved pileus) were harvested by severing the base just above the surface of the substrate with a sharp blade. Two rounds of mushroom harvests were made across all substrate types in the course of the experiment. To evaluate the growth performance of mushroom on different substrates, yield and biological efficiency were calculated. Accordingly, biological yield (g) was determined by weighing the whole cluster of fruiting bodies without removing the base of stalks, and economic yield (g) was determined by weighing all the fruiting bodies on a substrate after removing the base of stalks. Finally, biological efficiency (%) was calculated as follows:

$$\% BE = \frac{FWm}{DWs}*100\;\% ;$$

where, BE is Biological Efficiency (%); FWm is total fresh weight (g) of mushroom yield across all flushes, and DWs is substrate dry weight (g).

Then, analysis of variance (ANOVA) was computed using SPSS version 20, and mean values of all the parameters and the standard errors of each parameter were separated using LSD at 5 % level of significance.

## Results

### Time elapsed for mycelial running, pin-head formation and maturity of fruiting body

**Fig. 1:**
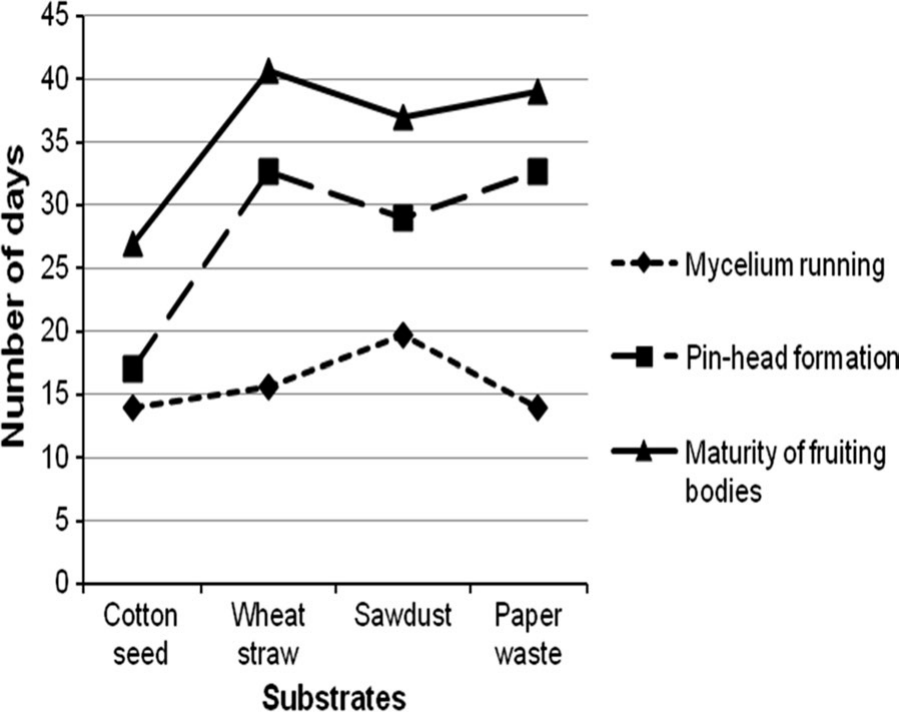
Time elapsed for mycelia running, pin-head formation and maturity of fruiting bodies of oyster mushroom under different substrates

Results on the overall time required for mycelial running, pin-head formation (primordium initiation), and maturity of fruiting bodies are illustrated in Fig. 1. Mycelial running is an extension and colonization of fungal hyphae throughout the substrate. The mycelial growth was faster on cotton seed and paper waste (14 days) than on wheat straw (15.67 days) and sawdust (19.67 days). The interim period of pin-head formation varied with substrates, ranging from 17 to 33 days after spawn seeding. Pin-head formation occurred quickly in cotton seed (17 days), followed by sawdust (29 days); while it took relatively longer time in wheat straw (32.66 days). Besides, the observation on the density of mycelia reveals that cotton seed and paper waste have higher mycelia density as compared to that of wheat straw and sawdust.

In a similar vein, the time required for maturity of fruiting bodies varied from 27 days (for cotton seed) to 40.67 days (for wheat straw). The cropping period for the other two substrates (sawdust and paper waste) was 37 and 39 days, which is more or less close to the maximum period recorded on wheat straw.

### Biological and economic yield

Results of the yield components (yield attributes) of oyster mushroom grown in each substrate are presented in Table 1. Accordingly, it was found that the product from paper waste has a relatively better growth in terms of diameter and thickness of the pileus, and diameter and length of the stipe. Moreover, the number of well-developed fruiting bodies was also recorded. It was observed that the number of fruiting bodies was significantly higher in the culture of cotton seed than the other substrates. The lowest number of fruiting bodies was recorded in sawdust. Matured fruiting bodies of oyster mushroom were harvested and weighed to determine biological and economic yield. Biological yield (g) was determined by weighing the whole cluster of the fruiting bodies without removing the base of the stalks, while economic yield (g) was determined after removing the base of the stalks. Results of the biological and economic yield are presented in Table 2.

**Table 1:** Yield attributes of *P. ostreatus* grown on different substrates

Parameters	Substrates			
	Cotton seed	Wheat straw	Sawdust	Paper waste
No of fruiting bodies	32.00^a^	18.30^b^	11.50^c^	18.64^b^
Pileus diameter (cm)	6.95	7.07	7.73	8.31
Pileus thickness (cm)	1.00	1.03	0.91	1.05
Diameter of stipe (cm)	3.11	3.21	4.74	4.85
Length of stipe (cm)	2.95	2.81	3.29	3.81

Mean values under the same category that bear different superscript letters are significantly different (α < 0.05)

**Table 2:** Biological and economic yield of *P. ostreatus* grown on different substrates

Substrates	Biological yield (g)	Economic yield (g)
Cotton seed	315.75^a^	277.30^a^
Paper waste	235.83^ac^	192.45^be^
Wheat straw	205.85^bc^	174.25^ce^
Sawdust	78.90^b^	64.33^d^

Mean values under the same column that bear different superscript letters are significantly different (α < 0.05)

As shown in Table 2, the use of different substrates brought about a significant (P < 0.05) effect on yield (biological and economic yield) of oyster mushroom. The largest yield was harvested from cotton seed, followed by paper waste; while, the least was obtained from sawdust. Further separation of the mean yields was also made using the LSD test to see whether there is significant yield difference among the substrates (Table 3).

**Table 3:** Separation of average yield values among treatments using LSD test

Treatments (I)	Treatments (J)	Mean difference (I–J) [Md±SE]	
		Biological yield (g)	Economic yield (g)
Cotton seed	Wheat straw	143.85^a^ ± 56.65	133.15^a^ ± 48.35
	Sawdust	261.68^a^ ± 56.65	247.77^a^ ± 48.35
	Paper waste	114.52 ± 56.65	114.95^a^ ± 48.35
Wheat straw	Cotton seed	−143.85^a^ ± 56.65	−133.15^a^ ± 48.35
	Sawdust	117.83 ± 56.65	114.62^a^ ± 48.35
	Paper waste	−29.33 ± 56.65	−18.20 ± 48.35
Sawdust	Cotton seed	−261.68^a^ ± 56.65	−247.77^a^ ± 48.35
	Wheat straw	−117.83 ± 56.65	−114.62^a^ ± 48.35
	Paper waste	−147.17^a^ ± 56.65	−132.82^a^ ± 48.35
Paper waste	Cotton seed	−114.52 ± 56.65	−114.95^a^ ± 48.35
	Wheat straw	29.33 ± 56.65	18.20 ± 48.35
	Sawdust	147.17^a^ ± 56.65	132.82^a^ ± 48.35

^a^The mean difference is significant at the 0.05 level

Accordingly, it was found that the biological yield from cotton seed was significantly different from the rest substrates, except paper waste at 5 % confidence level. For the economic yield, however, the mean values from cotton seed were significantly higher than all other treatments (substrates).

### Biological efficiency

Biological efficiency, which is used to evaluate the efficiency of substrate conversion in mushroom cultivation, was determined as ratio of the biological yield harvested to the dry weight of each substrate. As shown in Table 4, results of the biological efficiency varied significantly among the substrates used. The highest percentage of biological efficiency was obtained from cotton seed; while the least was observed in sawdust.

**Table 4:** Biological efficiency of *P. ostreatus* grown on different substrates

Substrates	Substrate dry weight (g)	Biological efficiency (%)
Cotton seed	425.70	74.17^a^
Paper waste	689.10	34.22^b^
Wheat straw	573.70	35.88^b^
Sawdust	810.90	9.73^c^

Mean values under the same column that bear different superscript letters are significantly different (α < 0.05)

## Discussion

### Time elapsed for mycelial running, pin-head formation and maturity of fruiting body

As per the finding of this study, the growth of *P. ostreatus* mycelia was relatively faster on cotton seed and paper waste as compared to the other substrates used (wheat straw and sawdust). On average, it took about 16 days for the mycelia to run on each substrate. This is comparable with other similar studies elsewhere. For instance, Onuoha et al. (2009) reported the completion of spawn running on paddy straw waste to be 15 days, while others reported it to be between 13 and 16 days using similar substrate (Patra and Pani 1995; Jiskani 1999). Similarly, Ahmed (1998) reported spawn running of *P. ostreatus* to be completed within 17–20 days on different substrates.

The variation in the number of days taken for a spawn to complete colonization of a given substrate is a function of the fungal strain, growth conditions and substrate type (Chang and Miles 2004). This variation could, in turn, be attributed to the variations in chemical composition and Carbon to Nitrogen ratio (C:N) of the substrates used (Bhatti et al. 1987). According to Oei (1996), mushroom mycelia require specific nutrients for its growth; the addition of supplements can, thus, increase mushroom yield through the provision of these specific nutrients.

Pin-head formation (premordium initiation) was observed following the invasion of substrates by mycelia growth. The time required for the formation of pin-heads is comparable with other similar studies elsewhere; e.g., Ahmed (1998) reported pin-head formation of oyster mushroom cultivated in different substrates to be between 23 and 27 days from spawning, while Fan et al. (2000) reported it to be 20–23 days. On the other hand, Shah et al. (2004) found that pin-heads appeared in about 6 days. Such variations in mycelia growth rate, colonization and primordial initiation have been observed when a mushroom species were grown on a range of substrates including sawdust, bagasse, and banana leaves (e.g. Vetayasuporn 2006; Islam et al. 2009; Birhanu Gizaw 2010).

It was, generally, observed from this study that the overall cropping period for oyster mushroom, in this case, the time elapsed between spawn seeding and harvesting (maturity of fruiting bodies), varied for each of the different substrates used; ranging from 27 days to 40.67 days. This implies that, with regard to the cropping period, cotton seed is the preferred substrate for early harvesting of yield of oyster mushroom. In this regard, other studies came-up with varying results of cropping periods. Khan and Ali (1981) reported a cropping period between 21 and 28 days using cotton seed, while Tan (1981) reported the harvesting time to be within a month using cotton waste. According to Khanna and Garcha (1981), however, it may take up-to 104 days to harvest yield from oyster mushroom grown on paddy straw. These variations in cropping periods may emanate from the variations in the growing environment (*controlled versus semi*-*controlled conditions*) and physiological requirements for mushroom cultivation, for instance, the constant temperature, humidity and light arrangements. In other words, the variation in cropping period among different substrates could emanate from variations in the time elapsed for formation of pinheads, maturation of fruiting bodies, period between flushes, number of flushes and yield, which in turn is affected by the nature of the substrates (Tan 1981).

### Yield and biological efficiency

Prior to determination of yield of oyster mushroom, measurements were made on the various yield components (yield attributes). It was observed that the yield components (yield attributes) of *P. ostreatus* were found to be affected by the use of different substrates though not significant. Paper waste resulted in a relatively better growth in terms of diameter and thickness of pileus, and diameter and length of stipe. On the other hand, the number of well-developed fruiting bodies was significantly higher in the culture of cotton seed than the other substrates. The lowest number of fruiting bodies was found in sawdust, while it was similar in wheat straw and paper waste. This could be indicative for the fact that the use of different substrates may affect the percentage of effective fruiting bodies formed.

The study confirmed that the use of different substrates brought about a significant (P < 0.05) effect on yield (biological and economic yield) of oyster mushroom. The largest yield was harvested from cotton seed, followed by paper waste; while, the least was obtained from sawdust. Similarly, the biological efficiency (BE) also varied significantly among the different substrates used. Variable ranges of BE have been reported when different lignocellulosic materials were used as substrates for cultivation of oyster (Liang et al. 2009).

In this study, the highest percentage of BE was obtained from cotton seed; the least was observed in sawdust. The performance of oyster growth and yield in sawdust substrate was minimal. This could be attributed to the fact that the lignocellulosic materials in sawdust are generally low in protein content and thus insufficient for the cultivation of mushrooms (Obodai et al. 2002). Therefore, sawdust substrate for mushroom production should undergo a period of composting to breakdown the cellulose and lignin components of the wood in order to release the essential materials for the establishment of mushroom mycelia. It may also require additional nitrogen, phosphate and potassium.

Besides, the mean comparisons (separated using LSD test) revealed that the biological yield from cotton seed was significantly different from the rest substrates, except paper waste at 5 % confidence level. For the economic yield, however, the mean values from cotton seed were significantly higher than all other treatments (substrates). Results of this study are in line with other studies elsewhere (e.g., Nout and Keya 1983; Choi et al. 1981), where cotton seed was identified as an important substrate for significant improvement in yield of oyster mushroom. The possible justification put forward is that cotton seed has fast decomposition rate, and hence, is accepted as a superior substrate over other lignocellulosic wastes, such as sawdust (Quinio et al. 1990).

Generally, the present study confirmed that oyster mushroom *(Pleurotus ostreatus)* can grow on cotton seed, paper waste, sawdust and wheat straw, with varying growth performances. Cotton seed followed by paper waste were identified as suitable substrates for oyster mushroom cultivation. Cotton seed produced a significantly higher yield and biological efficiency compared to the other substrates. It is also proved to be better in terms of mycelia density, time required for mycelia running, pin-head formation and development of fruiting bodies. Therefore, cotton seed can be recommended as the preferred substrate for oyster mushroom cultivation. In addition, paper waste can be used as alternative substrate given that the growth performance and yield of oyster mushroom was better in it next to cotton seed; this could also serve as a solution for utilization of the huge paper wastes available.

And yet, further studies need to be conducted on the potentials of various agricultural and industrial wastes on oyster mushroom cultivation, their economic feasibility and other related issues of mushroom (particularly oyster mushroom) to fully realize the multiple socio-economic and environmental significances of the mushroom industry in Ethiopia.
